# Rituximab Effectiveness and Safety for Treating Primary Sjögren’s Syndrome (pSS): Systematic Review and Meta-Analysis

**DOI:** 10.1371/journal.pone.0150749

**Published:** 2016-03-21

**Authors:** Francine Bertolais do Valle Souza, Gustavo José Martiniano Porfírio, Brenda Nazaré Gomes Andriolo, Julia Vajda de Albuquerque, Virginia Fernandes Moça Trevisani

**Affiliations:** 1 Department of Evidence-Based Medicine, São Paulo Federal University (UNIFESP), São Paulo, Brazil; 2 Department of Evidence-Based Medicine, São Paulo Federal University (UNIFESP), São Paulo, Brazil; 3 Department of Evidence-Based Medicine, São Paulo, Federal University (UNIFESP), São Paulo, Brazil; 4 Department of Evidence-Based Medicine, São Paulo Federal University (UNIFESP), São Paulo, Brazil; 5 Department of Evidence-Based Medicine, São Paulo Federal University (UNIFESP) São Paulo, Brazil; 6 Department of Rheumatology, Santo Amaro University (UNISA), São Paulo, Brazil; University of Bergen, NORWAY

## Abstract

**Background:**

Primary Sjögren’s Syndrome (pSS) is a systemic autoimmune disease that involves the exocrine glands and internal organs. pSS leads to destruction and loss of secretory function due to intense lymphoplasmacytic infiltration. Therapeutic options include mainly symptomatic and supportive measures, and traditional immunosuppressant drugs have shown no effectiveness in randomized trials. Rituximab (RTX) is a chimeric antibody anti-CD20 that leads to B cell depletion by diverse mechanisms. There is evidence that this drug may be effective for treating pSS. The objective of this systematic review was to evaluate Rituximab effectiveness and safety for treating pSS.

**Methods and Findings:**

We conducted a systematic review of RCTs published until December 2015, with no language restriction. We registered a protocol on *Plataforma Brasil* (40654814.6.0000.5505) and developed search strategies for the following scientific databases: MEDLINE, EMBASE, CENTRAL and LILACS. We included adults with established pSS diagnosis and considered the use of Rituximab as intervention and the use of other drugs or placebo as control. Four studies met our eligibility criteria: three with low risk of bias and one with uncertain risk of bias. The total number of participants was 276 (145 RTX, 131 placebo). We assessed the risk of bias of each included study and evaluated the following as primary outcomes: lacrimal gland function, salivary gland function, fatigue improvement and adverse events. We found no significant differences between the groups in the Schirmer test at week 24 meta-analysis (MD 3.59, 95% CI -2.89 to 10.07). Only one study evaluated the lissamine green test and reported a statistically significant difference between the groups at week 24 (MD -2.00, 95% CI -3.52 to -0.48). There was a significant difference between the groups regarding salivary flow rate (MD 0.09, 95% CI 0.02 to 0.16) and improvement in fatigue VAS at weeks 6 (RR 3.98, 95% CI 1.61 to 9.82) and week 16 (RR 3.08, 95% CI 1.21 to 7.80).

**Conclusions:**

According to moderate quality evidence, the treatment with a single RTX course in patients with SSp presents discrete effect for improving lacrimal gland function. Low-quality evidence indicates the potential of this drug for improving salivary flow. According to low quality evidence, no differences were observed in the evaluation after 24 weeks regarding fatigue reduction (30% VAS), serious adverse events occurrence, quality of life improvement and disease activity. With a very low level of evidence, there was no improvement in oral dryness VAS evaluation.

## Introduction

Primary Sjögren’s Syndrome (pSS) is a systemic autoimmune disease that involves the exocrine glands and internal organs. pSS leads to destruction and loss of secretory function due to intense lymphoplasmacytic infiltration [1,2]. Genetic, hormonal and external factors contribute to the development of this multifactorial disorder [1–3] which has a worldwide distribution and affects mainly women, in the ratio of 9:1. The incidence peak of pSS is between 40 and 60 years of age, although pSS can occur at any age [4,5].The first signs and symptoms of the pSS are usually nonspecific, therefore the diagnosis can take between 6 to 10 years to be established [6].

Around 50% of individuals with pSS may have systemic involvement including the pulmonary, renal, hepatic, pancreatic, vascular, central nervous and peripheral nervous systems [7]. Individuals with pSS present a large spectrum of alterations in laboratorial tests such as cytopenias, hypergammaglobulinemia, presence of anti-Ro/SSA and anti-La/SSB antinuclear antibodies, rheumatoid factor (RF), cryoglobulins and hypocomplementemia [8]. Therapeutic options include mainly symptomatic and supportive measures [9].

Rituximab (RTX) is a chimeric antibody anti-CD20 that leads to B cell depletion by diverse mechanisms. There is evidence that this drug may be effective for treating pSS [10]. However, the results of studies regarding RTX effectiveness are controversial, mainly due to different clinical manifestations [9–12].

The objective of this systematic review was to evaluate the effectiveness and safety of Rituximab for treating pSS.

## Methods

We conducted a systematic review of the literature in the Department of Evidence-Based Health of the São Paulo Federal University (UNIFESP). We registered the protocol on the *Plataforma Brasil* register of the Brazilian Ministry of Health (S1 Protocol).

### Search strategy

We developed search strategies including the following terms and synonyms: “rituximab”, “CD20 antibody rituximab”, “Mabthera”, “Roche brand of rituximab”, “rituxan”, “Hoffmann-La Roche brand of rituximab”, “IDEC brand of rituximab”, “Genentech brand of rituximab”, “IDEC-C2B8 antibody”, “IDEC-C2B8”, “Sjogren's Syndrome”, “Sjogren Syndrome”, “Sjogrens Syndrome”, “Syndrome Sjogren's”, “Sicca Syndrome” and “Syndrome Sicca”. We ran the search strategies until December 2015 with filters for RCTs in the following databases: Cochrane Central Register of Controlled Trials (CENTRAL), MEDLINE (via PubMed), EMBASE and LILACS.

### Study selection

Two authors (FBVS and GJMP) independently screened titles and abstracts and evaluated the eligibility of the identified studies. They read the studies classified as possibly eligible in full text and decided which studies to include in the review. In case of language barrier, the authors submitted articles to a qualified translator, and in case of eligibility disagreements, a third author (VMFT) made the final decision. We discarded the non-eligible studies due to specified reasons.

We included only randomized controlled trials (RCTs) and excluded cluster or cross-over trials. We selected RCTs with participants over 18 years of age and with an established pSS diagnosis according to the 2002 American-European Revised Classification Criteria [13]. We considered the use of Rituximab as intervention and the use of other drugs or placebo as control.

We considered the following primary outcomes: lacrimal gland function, evaluated through the Schirmer test, lissamine green or fluorescin test and VAS; salivary gland function, evaluated through salivary flow rate and VAS; and fatigue evaluated through the Functional Assessment of Chronic Illness Therapy-Fatigue (FACIT-F), VAS and the Profile of Fatigue and Discomfort (PROFAD). We also analysed the adverse events reported by authors.

We considered the following secondary outcomes: quality of life, measured through the Short Form-36 (SF-36) health survey or other validated instruments; disease activity, evaluated through the EULAR Sjögren’s Syndrome Disease Activity Index (ESSDAI) [14]; alterations in laboratorial variables (B lymphocyte, immunoglobulin, RF and B lymphocyte activating factor—BAAF—levels); and symptom perception, evaluated through the EULAR Sjögren’s Syndrome Patient Reported Index (ESSPRI) [15].

Two authors (FBVS and GJMP) extracted data from the studies through a standardized form with information about the participants, intervention, comparison, outcomes and characteristics. We assessed the risk of bias in each included study as high, low or unclear through the Cochrane Collaboration's tool for assessing risk of bias [16], which is structured in seven domains: random sequence generation; allocation concealment; blinding of participants and personnel; blinding of outcome assessment; incomplete outcome data; selective reporting; and other sources of bias.

### Data synthesis

We included the data assessed at week 24 from baseline on the Review Manager (Revman) 5.3.4 software. We analysed continuum data through mean difference and dichotomous data through risk ratio. We calculated all the effect measures with a 95% confidence interval. We assessed the statistical heterogeneity between studies through the I^2^ statistic [17] and considered the presence of significant heterogeneity for values superior to 50%.

We planned subgroup analyses considering the organ specific commitment, intervention protocol (cycle, dosage, comparison to other drugs), time of disease and age. We also planned sensitivity analyses considering the risk of bias and heterogeneity between studies and the statistical model used in the meta-analyses. We evaluated the quality of evidence through the GRADE approach.

## Results

**Fig 1:**
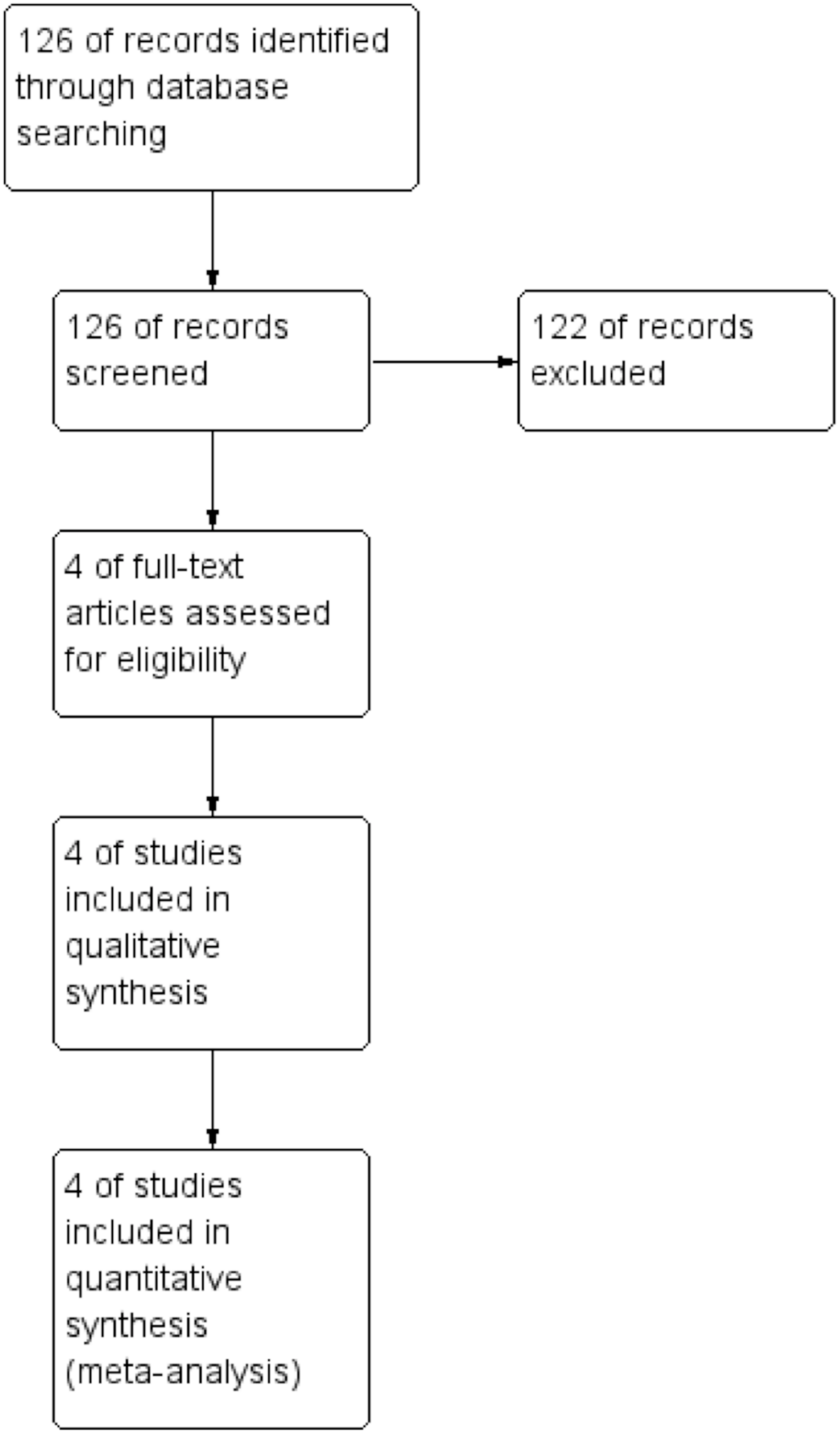
Flow chart of studies selection.

We identified 126 records and included four studies [18–20] in the quantitative analysis (Fig 1).

**Fig 2:**
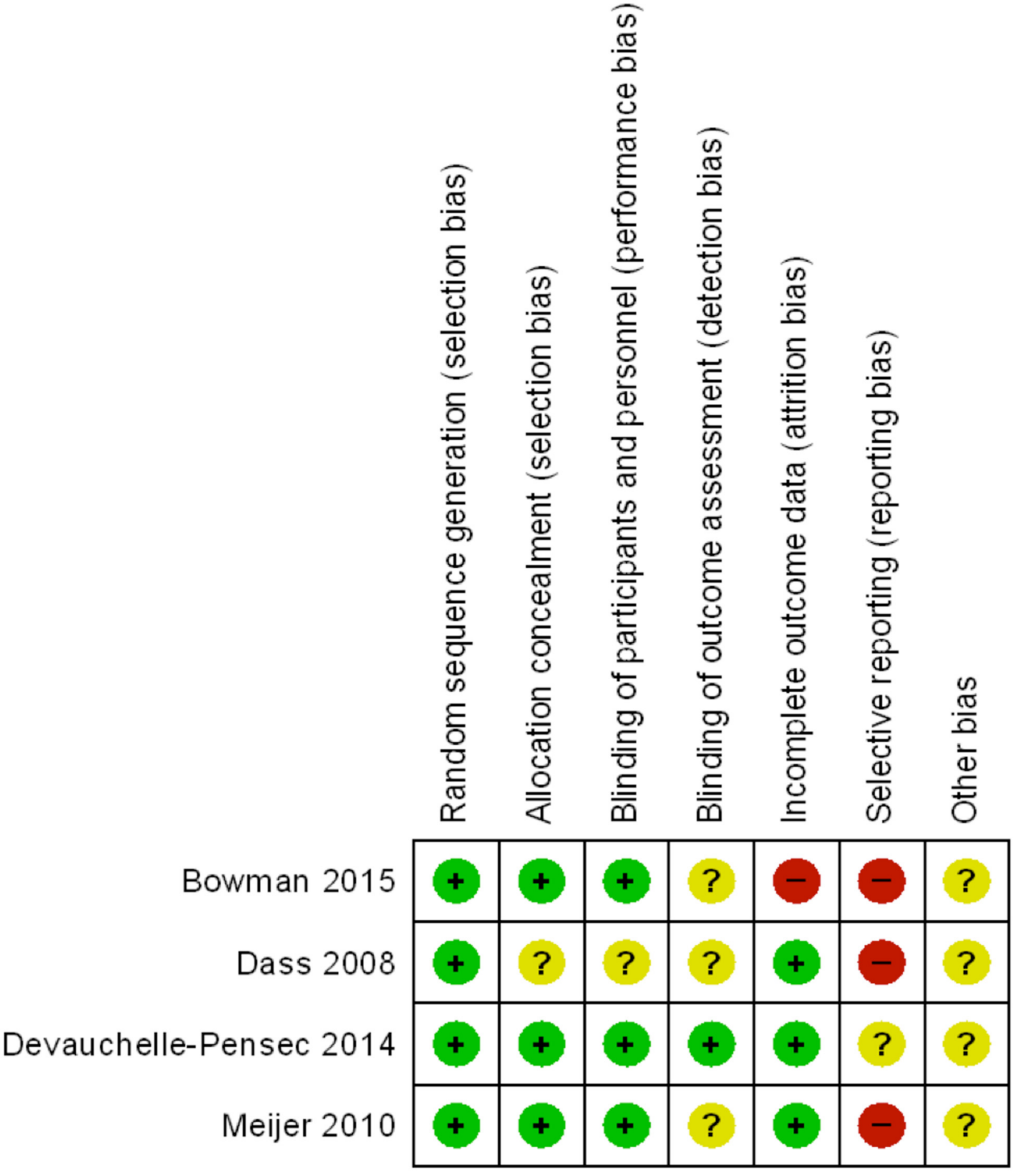
Risk of bias assessment of included studies.

We analysed each included study through the Cochrane Collaboration’s tool for assessing risk of bias (Fig 2).

The included studies totalized 276 participants: 145 in the RTX group and 131 in the control group (placebo). All included studies [18–21] randomly assigned patients to the RTX group (two 1g RTX infusions: day 1 and day 15, single course) or control group (placebo infusion: single course). We analysed data assessed 24 weeks from baseline.

### Primary Outcomes

**Fig 3:**
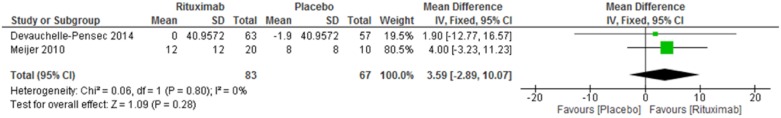
Rituximab X Placebo. Meta-analysis of the outcome Schimer test at week 24.

Three of the included studies evaluated lacrimal gland function through the Schirmer test [18–20]. Dass et al. [18] reported no changes but presented no results. Thus, we included only two studies [19,20] in the Schirmer test at week 24 meta-analysis. Meijer et al. [19] reported lacrimal gland function improvement in the RTX group since baseline until week 48. Yet, they found no differences between the groups in the Schirmer test. Devauchelle-Pensec et al. [20] found no effects of RTX in variables associated to dryness such as the results for lacrimal production in the Schirmer test. We performed a fixed-effect model meta-analysis of this outcome and observed no statistically significant differences between the RTX and placebo groups at week 24 (MD 3.59, 95% CI -2.89 to 10.07; Fig 3). We imputed the standard deviation data from Devauchelle-Pensec et al. [20] through *p* value and mean difference.

Meijer et al. [19] evaluated lacrimal gland function through the lissamine green test and found a significant difference between the RTX and placebo groups at week 24 (MD -2.00, 95% CI -3.52 to -0.48). Meijer et al. [19] also reported improvement of ocular dryness VAS scores in the RTX group since the baseline until week 48, whilst the scores in the placebo group only presented significant improvement after week 5. There was a significant difference in the average alteration of ocular dryness VAS between groups from baseline to week 24 (MD -27.00, 95% CI -46.28 to -7.72), week 36 (MD -24.0, 95% CI -44.5 to -3.5) and week 48 (MD -30.00, 95% CI -47.01 to -12.99).

**Fig 4:**
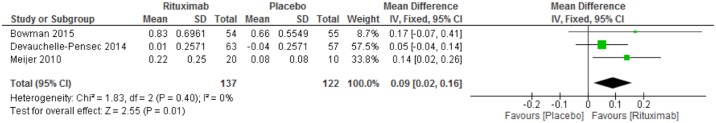
Rituximab X Placebo. Meta-analysis of the outcome salivary flow rate at week 24.

All included studies reported analysis of salivary flow data [18–21]. Dass et al. [18] reported no changes but presented no quantitative data, thus we excluded this study from the salivary flow rate at week 24 meta-analysis. Meijer et al. [19] reported improvement of salivary flow rate in the RTX group at week 24 (MD 0.14, 95% CI 0.02 to 0.26), whilst Devauchelle-Pensec et al. [20] and Bowman et al. [21] observed no significant differences between the RTX and placebo group at week 24 (MD 0.05, 95% CI -0.04 to 0.14 and MD 0.17, 95% CI -0.07 to 0.41, respectively). We performed a fixed-effect model meta-analysis of the outcome salivary flow rate and demonstrated a statistically significant difference between the groups in favor of the RTX group at week 24 (MD 0.09, 95% CI 0.02 to 0.16; Fig 4).

**Fig 5:**
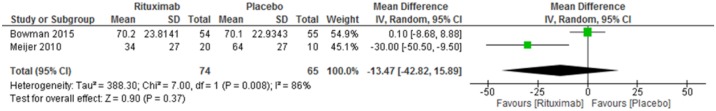
Rituximab X Placebo. Meta-analysis of the outcome oral dryness (VAS) at week 24.

Two [19–21] of the included studies evaluated oral dryness through VAS. Meijer et al. [19] reported improvement in the VAS score for all oral dryness symptoms in the RTX group. They found a statistically significant mean difference between the groups at week 24 (MD -30.00, 95% CI -50.50 to -9.50). We performed a random-effects model meta-analysis of this outcome and observed no statistically significant differences between the RTX and placebo groups at week 24 (MD -13.47, 95% CI -42.82 to 15.89; Fig 5). Bowman et al. [21] also evaluated oral dryness through VAS response rates (%) and found no significant differences between the groups (RR 0.93, 95% CI 0.45 to 1.93).

**Fig 6:**
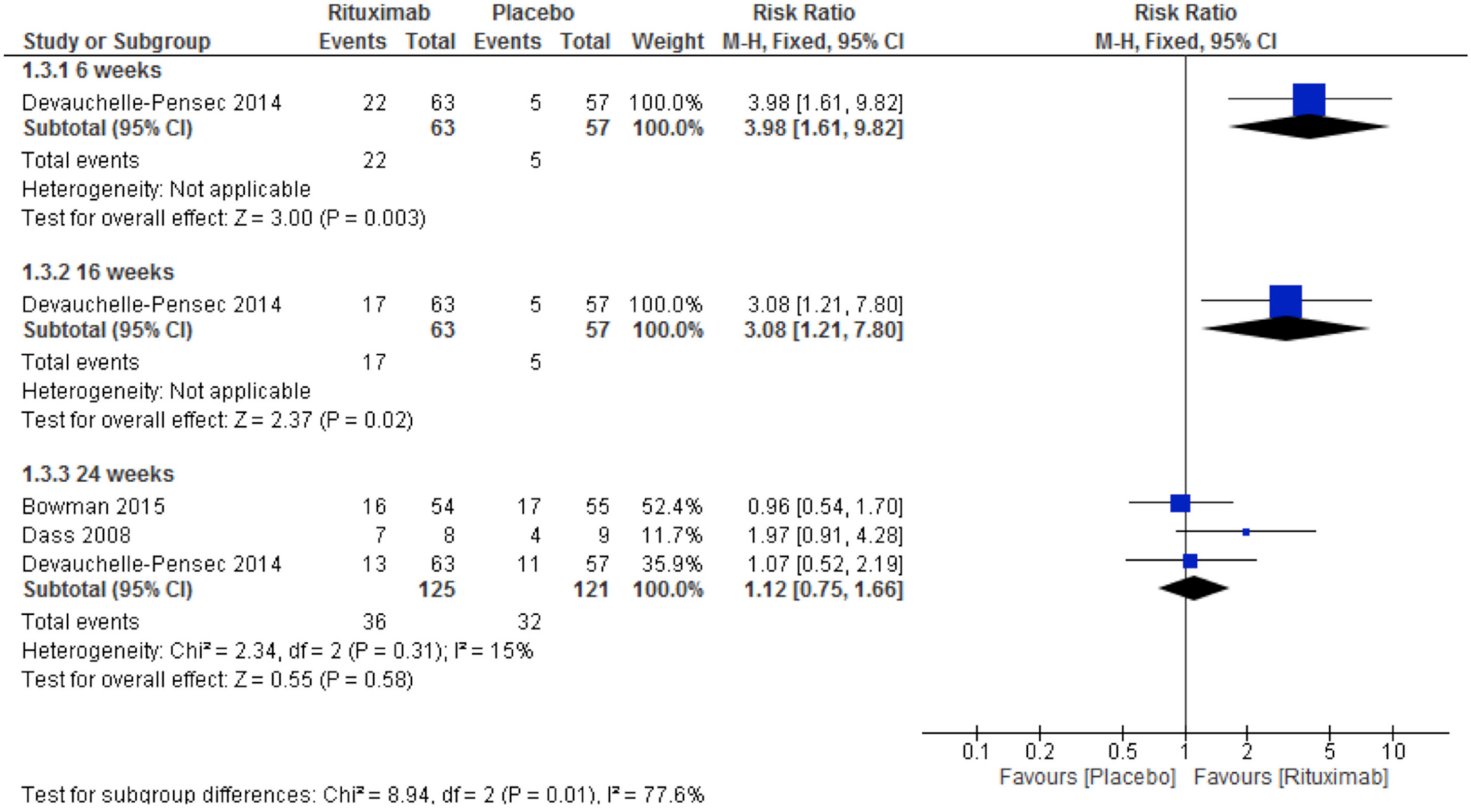
Rituximab X Placebo. Meta-analysis of the outcome fatigue VAS 30% improvement at week 24.

All included studies evaluated fatigue through VAS. Dass et al. [18], Devauchelle-Pensec et al. [20] and Bowman et al. [21] established up to 30% improvement as the primary endpoint. We found no significant differences between the groups in the meta-analysis of fatigue VAS 30% improvement at week 24 (RR 1.12, 95% CI 0.75 to 1.66; Fig 6). However, fatigue VAS results from Devauchelle-Pensec et al. [20] indicated a favorable response to RTX at week 6 (RR 3.98, 95% CI 1.61 to 9.82; Fig 6) and week 16 (RR 3.08, 95% CI 1.21 to 7.80; Fig 6). Bowman et al. [21], also reported fatigue outcome through VAS mean score (0-100mm, 100 = Severe) and found no significant differences between groups (MD 5.0, 95% CI -3.37 to 13.37). Only Dass et al. [18] evaluated fatigue through PROFAD and reported that there was a significant somatic fatigue domain improvement in the RTX group at week 24 (p = 0.009), but not in the placebo group (p = 0.087).

We evaluated the quality of evidence through the GRADE approach. The Schirmer test meta-analysis presented moderate quality. The salivary flow rate and fatigue VAS 30% improvement meta-analyses presented low quality evidence. The oral dryness VAS meta-analysis presented very low-quality evidence.

### Secondary outcomes

**Fig 7:**
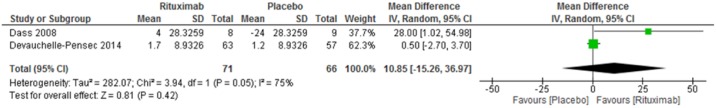
Rituximab X Placebo. Meta-analysis of the outcome SF-36 mental component summary at week 24.

Three of the included studies [18–20] evaluated quality of life through the SF-36 health survey but reported results differently. Meijer et al. [19] only reported the SF-36 total score and found no significant differences between the groups from baseline to week 48 (MD -5.00, 95% CI -17.15 to 7.15). Dass et al. [18] reported improvements in the mental health domain. Devauchelle-Pensec et al. [20] only described the mental and the physical component summary subscores and did not mention the SF-36 total score. They found no significant differences between the groups in the physical domain at week 24 (MD 0.60, 95% CI -16.90 to 18.10). Due to heterogeneity between studies, we performed a random-effects meta-analysis of mental health outcome at week 24 and found no significant differences between the groups (MD 10.85, 95% CI -15.26 to 36.97; Fig 7). This meta-analysis presented low-quality evidence according to the GRADE approach.

**Fig 8:**
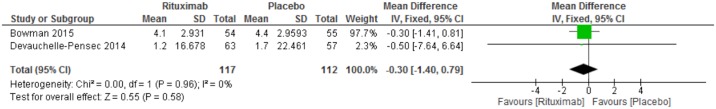
Rituximab X Placebo. Meta-analysis of the outcome disease activity assessed through the ESSDAI at week 24.

Devauchelle-Pensec et al. [20] and Bowman et al. [21] assessed disease activity through the ESSDAI. We performed a meta-analysis for this outcome and found no significant differences between the RTX and placebo groups (MD -0.30, 95% CI -1.40 to 0.79; Fig 8). According to the GRADE approach, this meta-analysis presented low quality evidence.

Dass et al. [18] demonstrated that in terms of laboratory results, there was a significant difference in RF reduction at week 24 favorable to the RTX group (MD 45, 95% CI 3.62 to 86.38). Devauchelle-Pensec et al. [20] mentioned they would analyse the RF as a secondary outcome, however, the result was not presented in the study.

Only Meijer et al. [19] described the analysis of B cells number. They observed an expressive decrease in the mean absolute number of B cells after the first RTX infusion and no significant alterations in the placebo group. They found statistically significant differences in the mean absolute number of B cells between the groups since baseline until weeks 5, 12, 24, 36 and 48 (MD -0.23, 95% CI -0.31 to -0.15).

Two studies evaluated immunoglobulin levels [18–20]. Dass et al. [18] reported a percentage reduction difference in favor of RTX (p = 0,05). Devauchelle-Pensec et al. [20] found a significant IgM reduction favorable to the RTX group (MD 0.30, 95% CI 0.13 to 0.47).

Only Bowman et al. [21] evaluated symptom perception through the ESSPRI and found no significant differences between the RTX and placebo groups (MD 0.50, 95% CI -0.19 to 1.19).

### Safety

All included studies reported adverse effects. Infusion-related reactions such as shiver, macular rash and purpura were more frequent in the RTX, as well as gastrointestinal, musculoskeletal and respiratory disorders. However, Meijer et al. [19] and Devauchelle-Pensec et al. [20] found similar infection rates between the RTX and placebo groups.

Meijer et al. [19] and Devauchelle-Pensec et al. [20] reported, respectively, that one and two RTX patients developed purpura within 15 days after infusion. Devauchelle-Pensec et al. [20] reported one occurrence in the placebo group.

Devauchelle-Pensec et al. [20] reported events such as shortness of breath, dry cough, sneezing or throat irritation in seven RTX patients, and found a significant difference between the groups in the proportion of patients with at least one respiratory disorder 24 hours after an infusion (p = 0.014). Only one of these events was considered serious and all patients improved after an infusion decrease or the treatment interruption. One patient in the placebo group had an asthma attack within 15 days after infusion [20].

Devauchelle-Pensec et al. [20] reported two cancer diagnoses in the RTX group during the investigations: one at day 7 from baseline (squamous cell carcinoma) and the other at day 38 (breast cancer; this patient died 1 year after inclusion in the study). One patient from the placebo group was diagnosed with superficial basal cell carcinoma 125 days after inclusion in the study [20].

Bowman et al. [21] reported occurrence of more adverse events in the RTX group than in placebo (275 Placebo vs 325 RTX). However, the adverse events considered serious were equal between groups (10 vs 10). The authors also reported just one serious infusion reaction (RTX) and one serious anaphylaxis (placebo).

**Fig 9:**
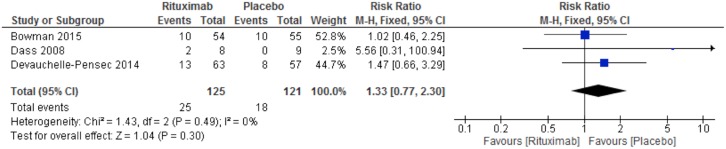
Rituximab X Placebo. Meta-analysis of the outcome serious adverse events at week 24.

Serious adverse events occurred in 25 participants of the RTX group [18,20,21] and in 18 participants of the placebo group [20,21]. We performed a fixed-effect meta-analysis for this outcome and found no significant differences between groups (RR 1.33, 95% CI 0.77 to 2.30; Fig 9). This meta-analysis presented low quality evidence according to the GRADE approach.

## Discussion

The number of published articles about the chimeric antibody anti-CD20 (rituximab) in the treatment of pSS has been growing over time. However, most identified studies are case reports or series of specific systemic manifestations. Of the studies found, only four met the inclusion criteria of our study, and one [21] was published in the form of preliminary results.Carubbi et al. [22] recently published a trial comparing RTX to immunosupressants and demonstrated the superiority of RTX. However, due to methodological aspects, this study could not be included in this review.Until recently, the treatment of pSS included only symptomatic and supportive measures. RCTs with traditional immunosupressants have shown no efficacy in the treatment of this multi-systemic disorder [23–27].The frequently serious pSS systemic features, the probability of evolution to lymphoproliferative disease [28] and the progress in the recognition of the role of cytokines (such as IFN-γ and IL-2) and the abnormal B cell activation established a possibility of new therapeutic measures [29–31].

Patients with pSS present B cell hyperactivity, which suggests an important role of these cells in the pSS pathogenesis [30–31]. RTX acts directly against these cells. Thus, RTX is considered a potential treatment for pSS.

There is still no consensus regarding the best time interval to evaluate the pSS treatment efficacy [20]. We considered a treatment period of 24 weeks for the outcomes analyses since this interval was common to all included studies.

Systemic manifestations were not evaluated in the studies and the follow-up period was short, therefore it was not possible to analyze bad prognostic factors of the disease. Our main results were regarding ocular gland function, salivary gland function and fatigue. Two studies [20,21] evaluated the disease activity and found no significant differences between the groups. The action of RTX in the reduction of RF and B cell number was demonstrated by Dass et al. [18] and Meijer et al. [19], respectively.

Patients with pSS present high incidence of non-Hodgkin lymphoma, mainly mucosa lymphomas [32,33]. The use of RTX, associated or not to chemotherapy, is an option for the treatment of this complication in pSS [32]. However, since the included studies did not report the presence of patients with lymphoma, it was not possible to conclude about this aspect.

A significant increase in the risk of adverse effects, mainly infusion reactions and respiratory disorders, was noted for participants allocated to RTX compared to those given placebo in all included studies. However, infection rates were similar between the groups in three studies: Dass et al. [18], Devauchele-Pensec et al. [20] and Meijer et al. [19]. Bowman et al. [21] reported that the occurrence of adverse events considered serious was equal between groups.

This review highlights the difficulty and inadequacy of research in pSS since that there are only a few randomized studies about RTX that compare the effectiveness of this drug to placebo or other drugs. The included studies presented similar endpoints, however the evaluation of outcomes still needs standardization. It was not possible to perform a subgroup or sensitivity analysis considering factors other than the model of effect used in the meta-analyses.

We evaluated only one treatment cycle since the assessment at week 24 was performed in all studies. Although Bowman et al. [21] administrated two RTX courses, they found no significant differences in any outcomes measured, except for salivary flow rate, as we have found in this study.

The number of participants included in this study was limited, even in the meta-analyses, and there was no efficacy analysis for different systemic manifestations. Regarding the benefits observed, only the outcome salivary flow demonstrated evidence of improvement, and RTX demonstrated itself to be safe since there were no differences in the presence of serious adverse events compared to the placebo group. For clinical practice, it is necessary to ponder the benefits and harms of this intervention in the treatment of pSS.

## Conclusion

According to moderate-quality evidence, the treatment with a single RTX course for patients with SSp presents discrete effect for improving lacrimal gland function. Low quality evidence indicates the potential of this drug for improving salivary flow. According to low quality evidence, no differences were observed in the evaluation after 24 weeks regarding fatigue reduction (30% VAS), serious adverse events occurrence, quality of life improvement and disease activity. With a very low level of evidence, there was no improvement of oral dryness VAS evaluation.

## Supporting Information

S1 PRISMA checklist (DOC)

S1 Protocol Registered on *Plataforma Brasil* (40654814.6.0000.5505). (DOCX)
