# Supplementary material for: Rituximab Effectiveness and Safety for Treating Primary Sjögren’s Syndrome (pSS): Systematic Review and Meta-Analysis
Source: PLoS One. 2016 Mar 21;11(3):e0150749. doi: 10.1371/journal.pone.0150749 (PMC4801187; doi:10.1371/journal.pone.0150749)
Supplement: S1 Protocol — (DOCX) [file pone.0150749.s002.docx]

**S1 Protocol. Protocol registered on *Plataforma Brasil* (40654814.6.0000.5505).**

Approved on 01/28/15.

**TITLE: Rituximab Effectiveness in Primary Sjögren’s Syndrome (pSS).**

**1. INTRODUCTION**

**1.1 Condition description**

Primary Sjögren’s Syndrome (pSS) is a systemic autoimmune disease that involves the exocrine glands and internal organs. Due to intense lymphoplasmacytic infiltration, pSS leads to the destruction and loss of secretory function. Genetic, hormonal and external factors contribute to the development of this multi-factorial disorder. With global distribution, women are mainly affected in the ratio of 9:1 and the peak incidence is between 40 and 60 years of age, although pSS can occur at any age. Early pSS manifestations are usually nonspecific; therefore, diagnosis can take from 6 to 10 years after early manifestations are established. Around 50% of patients may have systemic involvement including the pulmonary, renal, hepatic, pancreatic and vascular systems and both the central and peripheral nervous systems. pSS patients present a large spectrum of alterations in laboratorial tests such as cytopenias, hypergammaglobulinemia, presence of anti-Ro/SSA and anti-La/SSB antinuclear antibodies, rheumatoid factor (RF), cryoglobulins and hypocomplementemia. Therapeutic options include mainly symptomatic and supportive measures.

**1.2 Intervention description**

Rituximab (RTX) is a chimeric antibody anti-CD20 that leads to B-cell depletion by diverse mechanisms. There is evidence that this drug may be effective for treating pSS.

**1.4 Justification**

Recently some studies have suggested the use of Pilocarpina, Cevilemine, Hidroxicloroquina and Rituximab as main therapeutic approaches in pSS. However, the results of studies regarding RTX effectiveness are controversial, mainly due to different clinical manifestations.

**1.5. Objective**

The objective of this study is to perform a systematic review according to the Cochrane Collaboration’s methodology to evaluate the Rituximab effectiveness and safety for treating pSS.

**1.6. PICO**

P – Individuals with Primary Sjögren’s Syndrome.

I – Rituximab.

C – Immunosupressants, placebo or other interventions.

O – Lacrimal/salivary secretion improvement, fatigue and adverse events.

**2. Methods**

**2.1. Type of study**

This study is a systematic review of the literature with two independent evaluators and consensus meeting (Fig 1).

**
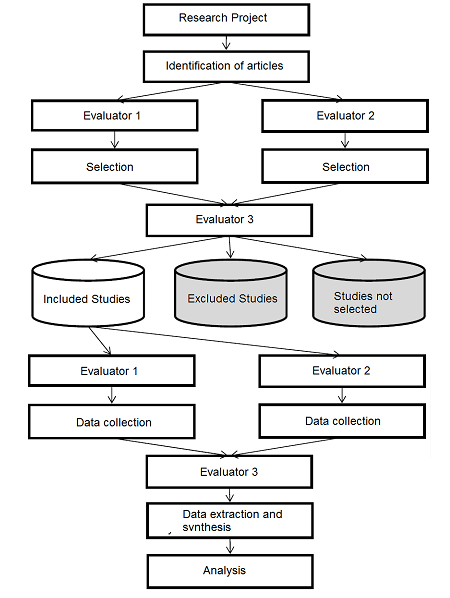
**

**Fig 1.** Research flowchart

**2.2 Location**

The present research proposal will be conducted in the Department of Evidence-Based Health of the São Paulo Federal University (UNIFESP).

**2.3 Sample**

**2.3.1 Inclusion Criteria**

*2.3.1.1 Regarding type of studies*

Randomized Controlled Trials.

*2.3.1.2 Regarding participants*

Participants over 18 years of age and with an established pSS diagnosis according to the 2002 American-European Revised Classification Criteria ^(13)^.

*2.3.1.3 Regarding intervention*

Rituximab (RTX) is a chimeric antibody anti-CD20 that leads to B-cell depletion by diverse mechanisms. There is evidence that this drug may be effective for treating pSS^(10)^.

*2.3.1.4 Regarding outcomes (primary and secondary)*

Primary Outcomes:

- Lacrimal and salivary gland function improvement through questionnaires, VAS and objective evaluation of salivary flow and oftlamological tests (Schirmer, lissamine green or fluorescin test).

- Fatigue improvement evaluated through the Fatigue Impact Scale (PROFAD), Functional Assessment of Chronic Illness Therapy Fatigue Subscale (FACIT-Fatigue), other validated questionnaires and fatigue VAS.

- Adverse events (by count of events).

Secondary outcomes:

Improvement of systemic manifestations through clinical evaluation, laboratory and functional tests and imaging:

- Disease activity evaluated through the EULAR Sjögren’s Syndrome Disease Activity Index (ESSDAI);

- Symptom perception evaluated through the EULAR Sjögren’s Syndrome Patient Reported Index (ESSPRI);

- Alterations in laboratorial variables (B lymphocyte, immunoglobulin, RF and B lymphocyte activating factor – BAAF – levels);

- Quality of life measured through the Short Form-36 (SF-36) health survey or other validated instruments**.**

**2.3.2 Data source**

Electronic Search:

We will run ran search strategies in the following databases: Cochrane Central Register of Controlled Trials (CENTRAL), MEDLINE (via PubMed), EMBASE and LILACS. We will locate ongoing researches through registers on the clinical trials database ClinicalTrials.gov.

Other sources:

We will contact authors regarding non detailed information in the methods of included articles. We will search manually in annals of conferences.

**2.3.3 Studies selection**

Two authors will independently screen titles and abstracts and evaluated the eligibility of the identified studies. They will read in full text the studies classified as eligible and discard the non-eligible studies. After reading eligible studies in full text, they will be excluded due to specified reasons or included in the review. There will be a consensus meeting to settle concordances in each selection phase.

**2.4. Data extraction and collection**

Two evaluators will extract data through a standardized form for collection of information about participants, interventions, comparisons and outcomes.

**2.5. Statistical Methods**

**2.5.1 Data type and measure of effects**

We will analyse continuous data through average difference or standardized mean difference. We will analyse dichotomous data through risk ratio, odds ratio and/or risk difference. We will calculate all the analyses with a 95% confidence interval.

**2.5.2 Analysis units**

We will analyse individuals. If we identify clusters studies, we will analyse them separately. We will analyse only the first part of cross over studies, before crossing. We will analyse adverse effects by count of events.

**2.5.3 Subgroup analysis**

We will perform analysis according to:

- Specific commitment;

- Intervention admnistration protocol (cyclic, dosage, comparison with other drugs);

- Time of illness;

- Age.

**2.5.4 Sensitivity analysis**

We will perform sensitivity analysis of the results according to:

- Studies risk of bias;

- Heterogeneity.

**2.5.5 Risk of bias assessment**

We will assess risk of bias in included studies through The Cochrane Collaboration’s tool for assessing risk of bias, structured in seven domains:

- Random sequence generation;

- Allocation concealment;

- Blinding of participants and personnel;

- Blinding of outcome assessment;

- Incomplete outcome data

- Selective reporting

- Other sources of bias.

**2.5.6 Publication bias**

We will evaluate publication bias through funnel plot if we have the necessary number of studies (n = 10).
